# Trends in Guideline-Driven Revascularization in Diabetic Patients with Multivessel Coronary Heart Disease

**DOI:** 10.3390/jcdd6040041

**Published:** 2019-11-18

**Authors:** Umme Rumana, Richard Kones, Montather O. Taheer, Mohamed Elsayed, Craig W. Johnson

**Affiliations:** 1 The University of Texas Health Science Center at Houston (UTHealth) School of Biomedical Informatics, Houston, TX 77030, USA; umme.rumana.mbbs@gmail.com (U.R.); craigandsuejohnson@gmail.com (C.W.J.); 2 Cardiology Section, Director, The Cardiometabolic Research Institute, Houston, TX 77054, USA; 3 The New York Institute of Technology, Old Westbury, New York, NY 11568, USA; mtaheer@nyit.edu (M.O.T.); melsay01@nyit.edu (M.E.)

**Keywords:** diabetes mellitus, coronary heart disease, coronary artery bypass graft, percutaneous coronary intervention, cardiovascular outcomes, coronary revascularization

## Abstract

In diabetes patients with chronic ≥3 vessel disease, coronary artery bypass grafting (CABG) holds a class I recommendation in the American College of Cardiology and American Heart Association (ACC/AHA) 2011 guidelines, and this classification has not changed to date. Much of the literature has focused upon whether CABG or percutaneous coronary intervention (PCI) produces better outcomes; there is a paucity of data comparing the odds of receiving these procedures. A secondary analysis was conducted in a de-identified database comprised of 30,482 patients satisfying the entry criteria. Odds of occurrence (CABG, PCI) were determined as the binary dependent variable in period 1, (17 October 2009 through 31 December 2011), and period 2 (1 January 2013 through 16 March 2015), before and after the 2011 guidelines, while controlling for gender, ethnicity/race, and ischemic heart disease as covariates. The odds of performing CABG rather than PCI in period 2 were not statistically significantly different than in period 1 (*p* = 0.400). The logistic regression model chi-square statistic was statistically significant, with χ_2_ (7) = 308.850, *p* < 0.0001. The Wald statistic showed that ethnicity/race (African American, Caucasian, Hispanic and Other), gender, and heart disease contributed significantly to the prediction model with *p* < 0.05, but ethnicity ‘Unknown’ did not. The odds of CABG versus PCI in period 2 were 0.98 times those in period 1 95% confidence interval (CI) = (0.925, 1.032), statistically controlling for covariates. There was no significant rise in the odds of undergoing a CABG among this dataset of high-risk patients with diabetes and multivessel coronary heart disease. Modern practice has evolved regarding patient choice and additional variables that impact the final revascularization method employed. The degree to which odds of occurrence of procedures are a reliable surrogate for provider compliance with guidelines remains uncertain.

## 1. Introduction

Coronary heart disease (CHD) is the leading cause of death in the United States in men, women, Hispanics, African Americans, and Whites [1]. According to current mortality rate data, CHD is the most common type of heart disease, killing one American approximately every 84 seconds [2].

Since 2015, life expectancy in the US has declined [3]. From 2006 to 2016, the annual death rate attributable to CHD fell by 32% and the actual number of deaths declined by 15%, while risk factor prevalence and intensity have arguably raised the overall burden in populations [4,5]. Some evidence suggests that the reduction in CHD mortality reversed in 2012, likely due to a rise in mortality related to the diabetes and obesity epidemics [6]. Presently, at least 48% of US adults have some form of clinical heart disease while 62% have subclinical CHD [2,5].

Diabetes mellitus type 2 (DM) is a major risk factor for CHD, increasing CHD mortality 2- to 4-fold as compared with those without DM [2]. Intensive oxidative stress and multiple disturbances in metabolic pathways are responsible for the aggressive, vigorous progression of atherogenesis in the presence of DM [7]. Although major steps have been identified, sufficient details to reverse the processes remain elusive [8]. Long incubation periods of both diseases assure that the molecular damage is advanced, and the lesions are diffuse at the time of presentation [9,10]. 

Two procedures are available to restore coronary arterial flow when pathology becomes critical or life-threating, each with a different design, distinct effects on the coronary vasculature, benefits, disadvantages, and outcomes. Coronary artery bypass grafting (CABG) via sternotomy became available in the 1960s after the introduction of machinery permitting cardiopulmonary bypass [11]. Techniques, safety, and outcomes improved, among which was thoracoscopic harvesting of the left internal thoracic artery, followed by minimally invasive and robotic surgery. The numbers of CABG performed has fallen from its peak in 2000 through 2018 [2,11,12]. 

Percutaneous coronary intervention (PCI), a less invasive catheter-based technique to compress lesions into the arterial wall, now typically followed by placement of a stent, began in 1977. The reduction in hospital stays, rapid recovery, outstanding success in acute obstructions, and low stroke rates increased popularity among physicians and patients alike [12]. Challenges included restenosis, thrombosis, and bleeding, which have been minimized by steady improvements in techniques and catheter and stent technology.

## 2. Why Is This Research Needed and Important?

In patients with multivessel coronary disease, coexistence of DM confers additional qualitative and quantitative risks that accelerate lesion development and portends poorer outcomes. Two decades ago, the National Heart, Lung, and Blood Institute indicated that CABG was associated with higher survival rates than PCI in patients with DM, based on the Bypass Angioplasty Revascularization Investigation (BARI) [13]. The major revascularization news was the emergence of new drug-eluting stents (DES) in the mid-2000s. From 2005 through 2010, a longitudinal trial—Future Revascularization Evaluation in Patients with Diabetes Mellitus: Optimal Management of Multivessel Disease (FREEDOM)—enrolled 1900 diabetic patients with multivessel CHD at 140 international centers [14]. Patients were randomly assigned to treatment with either CABG surgery or PCI using DES. Although the question of DES performance remains in flux, FREEDOM showed that following revascularization, “in the CABG group, the primary composite outcome of death, myocardial infarction, or stroke over 5 years was reduced by 7.9 percentage points, or a relative decrease of 30%, as compared with PCI (18.7% vs. 26.6%, *p* = 0.005)” [15]. Risk of all-cause mortality and myocardial infarction (MI) were both significantly reduced with CABG as compared with PCI with DES, but a higher risk of stroke was evident in the first 30 days after CABG. These findings confirmed what was previously believed [14,15]. The ACC/AHA issued guidelines in November 2011 advising that CABG produced better outcomes than PCI for patients with multivessel CHD generally, and specifically for diabetic patients [16]. This was a class I recommendation: CABG should be performed in patients with diabetes with multivessel (≥3) disease since the benefits outweigh the risk based on comparative effectiveness metrics.

In diabetic patients with multivessel disease, CABG is preferred over PCI with DES for optimal long-term survival and quality of life. Despite the 2011 guidelines and a large confirmatory study, results from the Get With The Guidelines National Registry trial show that the proportion of PCIs performed, at least in patients with diabetes and non-ST segment myocardial infarction, continued to be high, with just one-third of eligible DM patients with multivessel CHD and non-ST elevation myocardial infarction undergoing CABG [17].

The present study tested whether odds of performing CABG versus PCI procedures in diabetic patients with ≥3 vessel coronary disease differed during the 26.5 month period immediately before (period 1: 17 Octover 2009–31 December 2011) and beginning one year after (period 2: 1 January 2013–16 March 2015) the issuance of the ACC/AHA guidelines.

## 3. Hypothesis

In view of the above, we hypothesized that the odds of performing CABG versus PCI procedures would not surge in period 2, despite the guideline advisory and the FREEDOM study (which was the single largest study of such patients using contemporary optimum medical therapy, diagnostic technology, and DES).

Null hypothesis (H0): in patients with diabetes and multivessel CHD, the odds of undergoing a CABG rather than PCI were not higher in period 2, statistically controlling for gender, ethnicity, and ischemic heart disease. 

Alternative hypothesis (HA): in patients with diabetes and multivessel CHD, the odds of undergoing a CABG rather than PCI were higher in period 2, statistically controlling for gender, ethnicity, and ischemic heart disease.

Research question: is there a difference in odds of having CABG versus PCI performed in patients with diabetes with ≥3 vessel coronary disease from 17 October 2009 through 31 December 2011 (period 1), as compared with 1 January 2013 through the 16 March 2015 (period 2) in an extensive database? If ACC/AHA 2011 guidelines and FREEDOM recommendations were followed, and evidence and providers were responsible for the choice, then the odds of receiving a CABG during period 2 would have been higher than during period 1.

## 4. Methodology

Data source: The Cerner Health Facts^®^ Database captures and stores de-identified, longitudinal electronic health record patient data from 480 facilities in the United States, and then aggregates and organizes it into data sets for analysis [18]. Organization of the data permits tracking use of devices, diagnoses, and major procedures in hospitals and affiliated facilities where PCI or CABG is performed, with data included from 1 January 2008 to 16 March 2015. Allowing a one-year interval for dissemination and implementation of the ACC/AHA 2011 guidelines, our logistic regression analysis excluded data from 2012. We included a total of 30,482 patients for the logistic regression analysis in the present study. 

Inclusion criteria: we included patients who had diabetes during the two periods of interest and who either underwent PCI or CABG for the revascularization of three or more coronary artery obstructions visible on coronary arteriography.

Research design: the study using secondary data compares the odds of occurrence of revascularization procedures (CABG, PCI) as the binary dependent variable during time periods (period 1, period 2) before and after the 2011 guidelines, while controlling for gender, ethnicity/race, and ischemic heart disease as covariates.

**Figure 1:**
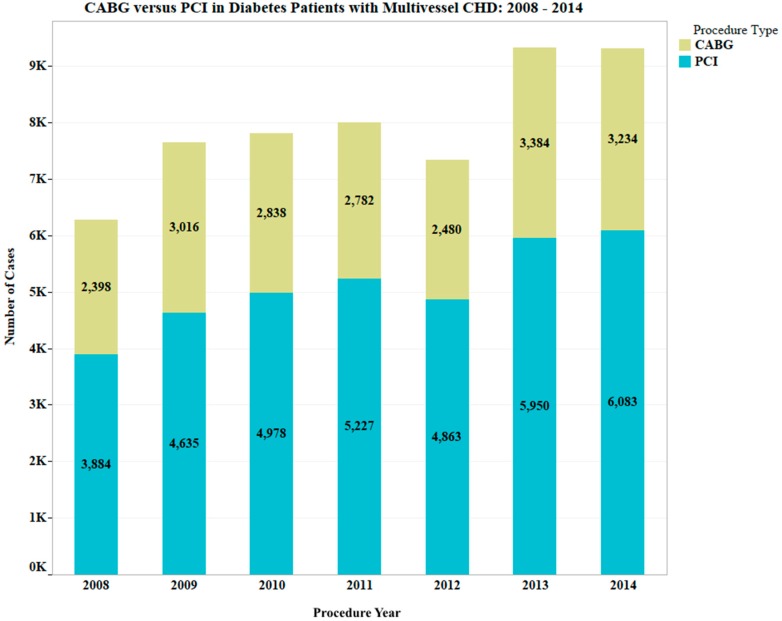
CABG versus PCI in diabetic patients with multivessel coronary heart disease (CHD) from 2008–2014. The graph includes patients from 2012 for comparison on an annual basis. As mentioned, this study did not include data from 2012 in the analysis and does not show patients analyzed in 2015.

Statistical analysis: given the dichotomous dependent/outcome variables (CABG, PCI; PCI as reference category), and continuous and categorical predictor variables, a logistic regression was performed [19] using time periods 1 and 2 as the levels of the independent variable under investigation while controlling for gender, ethnicity/race, and ischemic heart disease as covariates. Statistical analysis was conducted using IBM SPSS Statistics version 24. As indicated above, to allow a one-year interval for full implementation of ACC/AHA 2011 guidelines, the year 2012 was excluded from the logistic regression analysis. For the continuous period from 1 January 2008 through 31 December 2014, we graphed counts of patients (including those in 2012) from the database who underwent CABG or PCI, to show the recorded number of cases per procedure type annually (Figure 1). The analysis included only patients in periods 1 and 2.

Procedure: in November 2016, a central table of International Classification of Diseases, ninth edition codes for PCI and CABG involving ≥3 coronary artery lesions was constructed using SQL query and checked against the diagnosis codes for diabetes and coronary heart disease in the database. Those patients in the respective categories were gathered into our dataset.

## 5. Results

The odds of performing a CABG rather than PCI in period 2 were not statistically significantly different than in period 1 (*p* = 0.400). The logistic regression model chi-square statistic was statistically significant, with *χ^2^* (7) = 308.850, *p* < 0.0001. The Wald statistics showed that ethnicity (African American, Caucasian, Hispanic and Other), gender, and heart disease added significantly to the prediction model with *p* < 0.05, but ethnicity ‘Unknown’ did not.

The odds of CABG versus PCI in period 2 were 0.98 times those in period 1 (95% confidence interval (CI) = (0.925, 1.032)), statistically controlling for covariates (see Table 1).

The odds of performing a CABG in ethnicity category ‘Other’ (Asian, Asian/Pacific Islander, Biracial, Mid-Eastern Indian, Native American, Pacific Islander) were 0.68 times those in Caucasians, with *p* < 0.001 while statistically controlling for other covariates. Similarly, the odds of performing CABG in females were 0.69 times those in males, with *p* < 0.001 while statistically controlling for other covariates. The odds of performing a CABG in patients with heart disease were 2.26 times those without heart disease, statistically controlling for other covariates. Figure 1 shows that the number of cases per year remained higher for PCI throughout the years from 2008 to 2014. 

## 6. Discussion

The importance of accurate diagnosis and full treatment of CHD and DM in the United States cannot be overemphasized. Of the total 807,775 cardiovascular deaths in the United States in 2014, 45.1% were due to CHD [2]. From every point of view, the medical, personal, and economic burdens of this disease remain overwhelming. In 2010, there were approximately 954,000 inpatient PCIs and 397,000 CABG surgeries were performed. Within the perioperative and ensuing years, the disability and mortality associated with CHD is high after such treatments but differ according to the procedure and patient characteristics [2]. One of the goals of precision medicine is to provide the right treatment for the right patient at the right time. In this case, with different rates of successes and complications in the midst of a diabetes epidemic, individualizing the revascularization procedure challenges the most seasoned of practitioners.

The debate of whether to bypass CHD obstructions with surgical grafts (CABG) or engage in stent placement after lesion preparation (PCI) still continues. PCI advocates cite advances in stents, methodology recovery time, and lower perioperative stroke rates, while CABG advocates cite greater medium- and long-term survival, lower fatal and nonfatal event rates, less need for repeat revascularization compared to PCI, and possibly improved quality of life [20,21,22]. In patients with very high CHD risk, complex coronary lesions or diabetes, classical evidence supports the use of CABG [2,14,23,24,25]. The findings and commentary apply only to this patient population; in other applications, transcatheter techniques may be lifesaving and can provide advantages that surgery cannot offer.

In this analysis, the variation in odds ratio of performing a CABG versus PCI across periods 1 and 2 was not significant. Since the likelihood of having CABG performed in DM patients with ≥3 vessel CHD was not different after guidelines recommended CABG rather than PCI, the data provide no evidence that issuance of the ACC/AHA guidelines in November 2011 was followed by a significant change in the usage of CABG as compared with PCI. If providers fully controlled usage, compliance with the guidelines for the 30,462 patients in the present study would be expected to have resulted in significantly lower odds of PCI in period 2.

The odds of performing CABG in the ‘Other’ ethnic category were 0.63 times lower than in Caucasians. Several reasons may explain why more PCIs were performed in this group. Our study was not designed to explore them, but differing burdens of disease and disparities in care likely contributed. In addition, as per Table 1, the odds of performing a CABG in female patients was 0.69 times lower than in male patients, in part because women present with clinical heart disease some 8–10 years later than men, and in part because despite equal eligibility, women receive fewer cardiology services than men, particularly revascularization and CABG [26,27,28,29,30].

Recent data tend to confirm the current superiority of CABG in the study population [31,32,33,34,35,36,37]. One must be mindful that advances in technology, techniques, and materials occur rapidly. Given this state of flux, careful and frequent reevaluation of such views is prudent. A notable study included 11 randomized trials involving 11,518 patients [32]. This was the first adequately powered clinical trial able to detect a difference in all-cause mortality between CABG and PCI using stents, meaning bare-metal stents (BMS) and both generations of DES. Among the 3,051 patients who received only BMS, there was no statistically significant difference in five-year all-cause mortality between PCI and CABG (8.7% vs. 8.2%; Hazard ratio1.05; 95% CI 0.82–1.34). The use of BMS versus DES did not interfere with the overall findings (*p* for interaction = 0.53). The mortality difference between PCI and CABG groups was evident whether patients received first- versus newer-generation DES. In patients with DM, PCI was associated with a higher risk of all-cause mortality at five years compared with CABG (15.7% vs. 10.7%; HR 1.44; 95% CI 1.20–1.74). This relationship was maintained among patients with multivessel disease, with a five-year all-cause mortality of 11.5% for PCI and 8.9% for CABG (HR 1.28; 95% CI 1.09–1.49). In patients with both diabetes and multivessel disease, this difference was greatest (15.5% vs. 10.0%; HR 1.48; 95% CI 1.19–1.84). The investigators concluded that DM and multivessel/complex CHD are the most important drivers of adverse events, including mortality [32].

Bhatt, a Professor at Harvard and Executive Director of Interventional Cardiovascular Programs at Brigham and Women’s Hospital, wrote an editorial regarding the report. He noted that with each advance in PCI technology (balloon angioplasty, BMS, DES, use of catheter imaging with higher resolutions, and fractional flow reserve), calls have been made “to re-challenge CABG primacy for multivessel disease” [34]. However, as lesion complexity rose in patients with multivessel CHD, the mortality benefit of CABG over PCI increased [33]. “For patients with multivessel CHD and DM who are clinically and angiographically suitable for CABG or PCI, CABG is the clear choice” [34]. In those with multivessel CHD without DM, either approach could be considered; patient preference in addition to operator experience and talent should be key determinants. For patients with left main coronary artery disease without complex multivessel CHD with or without DM, PCI is an appropriate choice.

The follow-up study from the FREEDOM study (median 7.5 years) reported that the all-cause mortality rate was statistically significantly greater in the PCI/DES group than in the CABG group (24.3% deaths vs. 18.3%), leading to the conclusion that CABG is associated with lower all-cause mortality than PCI/DES [35,36]. Most interesting is the summary by the editorialist: “CABG targets the flow-limiting lesions as well as some non–flow-limiting lesions (in the 50-mm segment of the coronary artery that is bypassed) [37]. However, CABG is invasive and is associated with upfront morbidity and mortality with a prolonged recovery time although long-term mortality and quality of life (QOL) is improved. PCI targets only the flow-limiting lesions but has the advantage of being less invasive than CABG, with lower upfront morbidity and mortality, and shorter recovery time.” However, revascularization is much more complete with CABG, and according to Doenst et al. [25], the majority of MIs are generated by non–flow-limiting stenoses. The extent of repeat revascularization with PCI is appreciably greater as compared with CABG, and MI may be increased. Again, this pertains to patients with complex chronic multivessel CHD and DM, not other clinical settings. Not all patients are eligible for either approach; often the individual scenario dictates which the better match would be, and other times the patient chooses PCI solely according to his/her personal preference.

## 7. Limitations and Delimitations

The study design included a period before and a period after the issuance of the ACC/AHA 2011 guidelines and the FREDDOM study. Data from 2012 were excluded from our dataset, allowing one year for full implementation to occur. In addition, the dataset did not extend beyond 16 March 2015, possibly due to uncertainties about the implementation of the ICD-10 codes. For this reason, the database used, limited to ICD-9 codes, somewhat truncated our dataset.

Further, since diagnosis codes in the CHD series were not combined with procedure codes, the primary extraction method used the procedure type (CABG, PCI). It is possible, therefore, that these procedures were performed for conditions other than CHD. Given the prevalence of CHD and the relative rarity of, for instance, malformations, it is believed that the potential impact upon our findings is minimal.

In this study, we asked: did interventional cardiologists and surgeons follow the ACC/AHA 2011 guidelines and FREEDOM trial recommendations in period 2, as compared with period 1? Since the recommendations (CABG or PCI) for many variables (gender, age, ethnicity, high or low left ventricular function, number of lesions ≥3, obstruction in the important left anterior descending artery, and others) are the same, this study does not seek the answer to this question according to, for instance, severity of disease or risk of post-intervention survival for each CABG or PCI category. Moreover, especially when newer techniques, methods, and materials, and improved risk scores become additional variables, available high-quality data becomes sparse. Even though differences in post-intervention survival are anticipated, our present study was not designed to compare the results of the two procedures, but only whether the relative frequencies of the procedures changed. Nonetheless, a clear understanding of what factors enter into decision-making is essential. The data elicit two relevant additional questions: first, what has occurred during the intervening period since 2014, partially addressed above? Second, from the data regarding procedure frequencies and calculated odds, with what confidence can one conclude that providers did not follow guidelines? For instance, if patients select PCI rather than CABG, following patients’ wishes would be within guidelines-directed care. In a secondary analysis, without this information and related details, the recording of another PCI instead of CABG in such patients could be considered as a procedure not conforming to guidelines.

A recent study used rating, ranking, standard gamble, willingness to pay, and discrete choice experiments to investigate patients’ preferences and trade-offs in evaluating CABG versus PCI [38]. These authors considered 12% of candidates were eligible for either procedure. Patient decisions were influenced by the complications documented in clinical trials, variables sometimes not included in comparisons of outcomes, and additional factors, such as postoperative pain, mediastinitis, postprocedural angina, length of stay, depression, scar formation, pneumonia, pseudoaneurysm, renal failure, bleeding, long term need for antiplatelet agents, medical side effects, longevity and “cure.” Of the six main studies included, the results were: (i) PCI was preferred (80%) relative to CABG (19%); (ii) stroke was weighed the most by patients, followed by repeat PCI, while physicians rated death most; (iii) MIs and death were weighed heavily; (iv) stroke was considered worse than death by some patients; (v) repeat PCI >28% (for original PCI) influenced decisions strongly; and (vi) patients preferred PCI over CABG even when risk of death with PCI was double, and the risk of repeat PCI was over triple the corresponding CABG risks. Based upon these and prior data, the authors concluded that the guideline recommendations do not reflect endpoints that adequately represent those of patients [38,39].

There has been growing interest in robotic and minimally invasive CABG surgery and in hybrid procedures. The goal is to improve patient recovery time and provide better patient experiences. Hybrid coronary revascularization (HCR) combines surgical bypass with PCI during the same or in staged procedures. An essential and effective component of CABG is grafting from internal thoracic arteries into the left anterior descending system [40]. This affords protection against future obstructions, increasing long-term patency of the graft and enhancing long-term outcomes. At the same time, PCI offers advanced stent technology with lowered rates of thrombosis and restenosis. Hybrid procedures appear attractive since they are less invasive than CABG, preserve the essence of bypass, and may provide less discomfort, faster recovery and discharge [41]. One could anticipate that when presented to patients, the dynamics of their decision-making might be clarified and less onerous. Actual HCR procedures across centers varies, and techniques and outcomes compared to PCI and CABG require further research [11,12].

## 8. Conclusions

Results from 30,482 diabetic patients who either underwent PCI or CABG during the 26.5 month periods immediately before and beginning one year after the issuance of the ACC/AHA November 2011 guidelines showed no evidence of a significant change in usage of CABG as compared with PCI. Our data suggest that, in this particular dataset and during the time periods studied, CABG lagged behind PCI in diabetic patients with multivessel disease. The preponderance of literature supports shared decision-making with patients so that they are empowered to contribute personal preferences and their own weights to the risk/reward balance. In addition, modern revascularization teams now include a medical cardiologist, an interventionist, and a cardiac surgeon. This practice, a “multidisciplinary heart team” approach included in the ACC/AHA 2011 guidelines, represents a meaningful advance in revascularization decision-making in patients with DM [42], and may alone reduce adverse cardiovascular events [43].

Future studies should not only reexamine our findings, but hopefully enroll a sufficient number of participants to address the unanswered questions about the procedures of choice in each subgroup with explanations. Are patients choosing PCI after a complete decision-making discussion with providers or the heart team? To what extent do providers actually influence the final choice, particularly in view of the current milieu of medical practice? Are fears of stroke, higher invasive nature, post-operative death, or other factors inordinately increasing patient preference for PCI? If so, in which clinical setting is this most significant, and what are realistic, evidence-based solutions? Are cultural beliefs, costs, or insurance coverage responsible?

Finally, lifestyle improvements as part of primordial, primary, and secondary prevention are uniformly recommended to optimize all revascularization outcomes. Correcting the underutilization of cardiac rehabilitation represents low-hanging fruit available to clinicians [6]. Interdisciplinary training provides a multi-pronged approach to cardiovascular care that uniquely matches contemporary patient needs with high potential [44].

Overall, continued persistence and investigation is essential to come closer to the goal of individualized, cost-effective, and optimal care of high-risk individuals with diabetes and advanced coronary heart disease.

## Figures and Tables

**Table 1:** Odds of performing coronary artery bypass grafting (CABG) versus percutaneous coronary intervention (PCI) in period 2 compared with period 1, controlling for covariates.

	*B*	S.E.	Wald	*df*	*p*	Odds Ratio	95% CI for Odds Ratio	
							Lower	Upper
Ethnicity			63.32	4	<0.001			
African American ^a^	−0.08	0.04	4.01	1	0.045	0.92	0.85	1.00
Hispanic ^a^	−0.30	0.15	3.89	1	0.049	0.74	0.55	1.00
Other ^a^	−0.47	0.06	58.74	1	<0.001	0.63	0.55	0.71
Unknown ^a^	−0.07	0.09	0.65	1	0.421	0.93	0.78	1.11
Gender ^b^	−0.38	0.03	155.88	1	<0.001	0.69	0.65	0.73
Heart Disease ^c^	0.81	0.11	54.39	1	<0.001	2.26	1.82	2.81
Period 2 vs. Period 1 ^d^	−0.02	0.03	0.71	1	**0.400**	**0.98**	0.92	1.03
Constant	−1.88	0.11	285.77	1	<0.001	0.15		

*B* = regression coefficient, S.E. = standard error, *df* = degrees of freedom, *p* = significance level, CI = confidence interval, a. reference category = Caucasian, b. reference category = Male, c. reference category = absence of heart disease, d. reference category = Period 1; dependent variable (CABG, PCI; PCI as reference category).
